# Periodontal and Peri-Implant Diseases and Systemically Administered Statins: A Systematic Review

**DOI:** 10.3390/dj9090100

**Published:** 2021-09-04

**Authors:** Federica Di Spirito, Luigi Schiavo, Vincenzo Pilone, Antonio Lanza, Ludovico Sbordone, Francesco D’Ambrosio

**Affiliations:** Department of Medicine, Surgery and Dentistry “Schola Medica Salernitana”, University of Salerno, Via S. Allende, Baronissi, 84081 Salerno, Italy; lschiavo@unisa.it (L.S.); vpilone@unisa.it (V.P.); alanza@unisa.it (A.L.); lsbordone@unisa.it (L.S.); f.dambrosio6@studenti.unisa.it (F.D.)

**Keywords:** periodontitis, peri-implantitis, statins, simvastatin, hyperlipidemia, periodontal disease

## Abstract

Hyperlipidemia is a well-recognized risk factor for cardiovascular disease, which, in turn, acts as a contributory factor in periodontitis development. Periodontitis has been associated with benign tumors and cancers and withseveral disorders, including hyperlipidemia. Correspondingly, periodontal treatment may exert a positive effect on lipid metabolism, although opposite evidence has also been reported. As a counterpart, the therapy for hyperlipidemia, conventionally based on statins, has been proposed to positively affect periodontal conditions, mainly due to statin pleiotropic effects, reducing periodontal inflammation and promoting osseointegration. Therefore, the present systematic review aimed to evaluate, in subjects with untreated periodontitis and peri-implant disease (Population), the effect of routine systemically administered statins (Intervention), compared to non-statin use (Comparison), on periodontal parameters around natural teeth and implants (Outcome). Discordant results were found in periodontal parameters, and the current lack of such data related to peri-implant tissues and to alveolar bone loss highlights the need for further studies on the topic, potentially paving the way for a more comprehensive approach to periodontitis and peri-implantitis management. Indeed, the validation of the beneficial effect provided by systemically delivered statins on periodontal and peri-implant tissues may direct recall scheduling, predict response to therapy and, therefore, guide treatment strategies of periodontal and peri-implant treatments in statin users.

## 1. Introduction

Hyperlipidemia is characterized by an increase in triglycerides (TGs), total cholesterol and low-density lipoprotein (LDL) serum levels, coupled with a decrease in blood concentrations of high-density lipoprotein cholesterol (HDL) [1]. Hyperlipidemia is a well-recognized risk factor for cardiovascular disease [2], which, in turn, acts as a contributory factor in periodontitis development [1]. 

Periodontitis is a chronic inflammatory disease initiated by periodontal pathogens in the bacterial biofilm that is subsequently sustained by periodontal tissues inflammation, leading to the disruption of the anatomical structures supporting the teeth, alveolar bone loss and, eventually, tooth loss [3,4,5,6,7]. Periodontal disease has been associated with several benign tumors and colorectal, breast and prostate [8,9] cancers, as well as a multitude of systemic disorders, including diabetes [10], inflammatory bowel disease [11], atherosclerosis, rheumatoid arthritis [12,13], obesity [14] and hyperlipidemia [1].

Correspondingly, periodontal treatment in hyperlipidemic periodontal subjects has been reported to improve serum lipid concentrations, decreasing total cholesterol and LDL and increasing HDL [15,16], and to reduce proinflammatory cytokines levels, such as C Reactive Protein (CRP), Tumor Necrosis Factor-a (TNF-a) and Interleukin-1 (IL-1) [17]. However, contrasting evidence does not support such a positive effect of periodontal non-surgical therapy on lipid metabolism [18]. 

As a counterpart, the therapy for hyperlipidemia has been proposed to positively affect periodontal conditions [19]; such therapy is conventionally based on statins [20], which inhibit the 3-hydroxy-3-methylglutaryl coenzyme A reductase, implicated in the synthesis of cholesterol [21], and are currently administered to the 83% of the subjects under cholesterol-lowering medications [22]. Beyond being used to treat hyperlipidemia and arteriosclerosis, statins are usually administered to prevent coronary artery diseases and, due to their effectiveness, safety, tolerability and low cost [23], their use has raised over the last years, covering about the 20–25% of the population over 40 years of age [22]. Some authors have described a positive effect exerted by statins on periodontal subjects [24,25], although opposite evidence was also reported by other authors [26,27], maybe due to the multiplicity of statin type and route of administration (systemic/local), as well as to the heterogeneity of periodontitis severity and treatment (non-surgical/surgical) analyzed in different studies [28]. 

Similar to periodontal tissues, peri-implant ones havealso been proposed to be influenced by statin intake. In more detail, both locally and systemically delivered statins seem to positively affect osseointegration, secondarily to the reduction in osteoclastic activity, on the one side, and to the induction of osteoblast differentiation, bone apposition and angiogenesis on the other side, which have been demonstrated in preclinical and animal studies [29]. 

Previous systematic reviews [20,28,30,31], including preclinical and animal studies, were focused on the effect of statins, mainly locally delivered ones, on outcomes of periodontitis treatment, without considering comorbidities,other pharmacological treatments and preexisting severity of periodontal tissues disruption. 

Given these considerations, the present systematic review aimed to evaluate, in subjects with untreated periodontitis and peri-implant disease (Population), the effect of routine systemically administered statins (Intervention), compared to non-statin or placebo use (Comparison), on periodontal parameters around natural teeth and implants (Outcome), including clinical trial, longitudinal, case–control and cross-sectional studies.

## 2. Materials and Methods 

### 2.1. Protocol 

The present study was conducted in compliance with the Preferred Reporting Items for Systematic Reviews and Meta-analyses (PRISMA) statement [32], freely available online: http://www.prisma-statement.org/ (accessed on 20 July 2021). 

Question formulation, as well as search strategies definition and study selection criteria identification, were carried out according to the PICO model [33] (https://linkeddata.cochrane.org/pico-ontology; accessed on 20 July 2021). The question of the present systematic review was “Do systemically delivered statins positively affect periodontitis and peri-implantitis?”, focusing:

P—Population: subjects with untreated periodontitis and peri-implant disease without comorbidities potentially affecting their periodontal/peri-implant status and not under drugs affecting lipid nor bone metabolism;
I—Intervention: effect of routine use of systemically delivered statin;
C—Comparison: no systemically delivered statin use/placebo administration;
O—Outcome: periodontal parameters around natural teeth and implants.

### 2.2. Information Sources and Search

An electronic search was conducted without dates of coverage restrictions, till June 2021, on Medline (PubMed) and Scopus databases, using the following keywords, combined by Boolean operators:

1. Statin/statins;
2. OR simvastatin/atorvastatin/cerivastatin/fluvastatin/lovastatin/pitavastatin/pravastatin/ rosuvastatin;
3. OR hydroxymethylglutaryl-coa reductase/hmgcoa reductase inhibitors;

AND

1. Periodontitis;
2. OR periodontal health/diseases/inflammation/pocket/lesion(s)/treatment/therapy/debridement/defect/parameters;
3. OR peri-implantitis;
4. OR peri-implant tissues/health/diseases/inflammation/mucositis/pocket/lesion(s)/treatment/therapy/debridement/defect/parameters;
5. OR CAL (acronym of Clinical Attachment Level);
6. OR PPD (acronym of Periodontal Probing Depth);
7. OR BoP (acronym of Bleeding on Probing);
8. OR guided periodontal tissue/guided bone regeneration;
9. OR crevicular cytokines/IL-1B/TNF-a/IL-6/IL-8.

### 2.3. Study Selection Process

Study selection was conducted by two independent reviewers (FDS, FDA), and any discrepancy was solved by discussion.

Title and abstract screening were accomplished for all the records identified through database search.

Full-text reading was performed for abstracts reporting ambiguous methods and for articles considered eligible for the present systematic review, based on the inclusion/exclusion criteria, reported in Table 1. 

References reported in the eligible articles were checked, and an additional literature search was carried out. Neither manual search nor contact with the corresponding author for online unavailable full-texts were performed. 

### 2.4. Data Collection Process and Items

Data extraction and collection were performed twice by two independent authors (FDS, FDA) in a dedicated form, developed from those proposed for intervention reviews on RCTs and non-RCTs [34], also available online: https://dplp.cochrane.org/data-extraction-forms (accessed on 20 July 2021). No additional processes were carried out to obtain nor confirm data from investigators. 

The following variables were recorded for each selected study: source (first author, year and journal of publication, complete article citation, fundings); study design; participants (sample size, age and gender, smoking habit, comorbidities, treatments and procedures); intervention (statin type, duration, dosing); comparison (no statine use, placebo); periodontal outcomes (clinical, radiographic and other periodontal parameters); consideration(s) and conclusion(s).

### 2.5. Risk of Bias Assessment for Individual Studies

Risk of bias in individual studies was assessed at the study level by two independent reviewers (FDS, FDA), and discrepancies were solved by discussion. 

The Cochrane Collaboration’s tool for assessing risk of bias [34] was employed to analyze the following six domains of bias and the related items in randomized clinical trials:

- Selection (random sequence generation, allocation concealment);
- Performance (blinding of investigators and participants);
- Detection (blinding of outcome assessment);
- Attrition (incomplete outcome data);
- Reporting (selective outcome reporting);
- Others.

The risk of bias was previously recorded for all of the items, as extensively shown in the risk of bias table. For each item, in case of sufficient data, the risk of bias was defined as “low” (-), therefore unlikely to seriously alter the results; for missing data, the risk of bias was declared as “high” (+), consequently, capable of serious alteration of the results; for insufficient information, the risk of bias was considered as “unclear” (?), raising doubts on study results. 

Subsequently, the overall risk of bias within a trial was assessed, and it was defined as “low” if all items and domains were already defined as at low risk of bias and as “unclear” or “high” with at least one item formerly judged as unclear or high, respectively.

The ROBINS-I tool was employed, instead, for assessing the following seven domains of bias of non-randomized studies of the effects of interventions [35,36], related to:

- Confoundingvariables;
- Selection of participants;
- Classification of interventions;
- Deviations from intended interventions;
- Missing data;
- Measurement of outcomes;
- Selection of the reported result.

## 3. Results

### 3.1. Study Selection 

**Figure 1:**
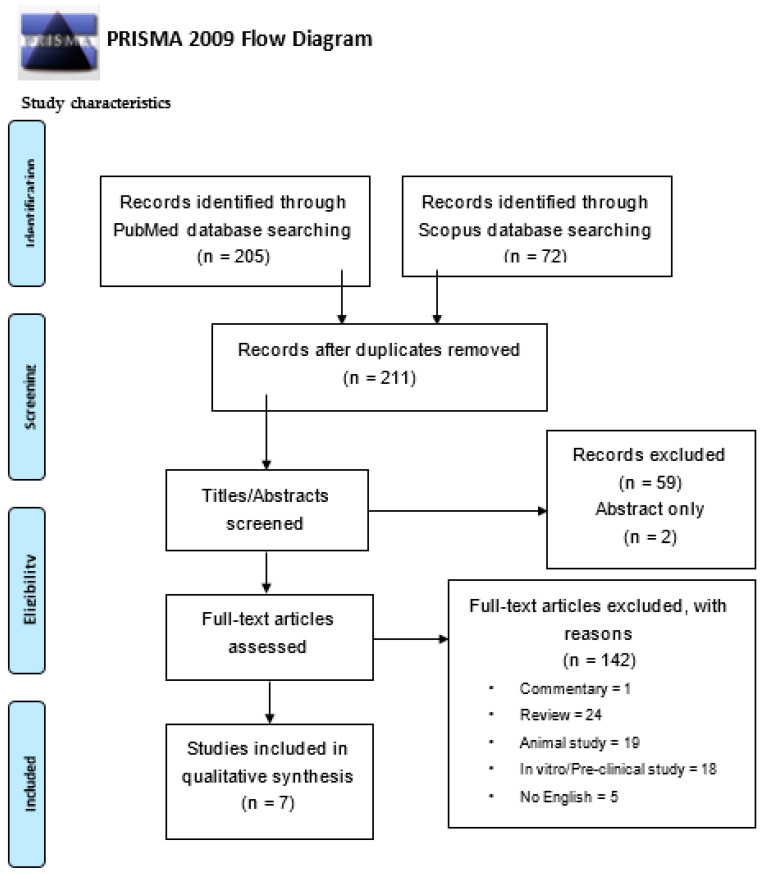
Study selection flow-chart.

The study selection flow-chart, illustrated in Figure 1, shows that, through the electronic search, a total of 277 potentially relevant titles/abstracts were initially retrieved: specifically, 205 from PubMed and 72 from Scopus databases, respectively. Subsequently to duplication elimination, 213 potentially pertinent title/abstracts were identified, comprising 202 articles concerning periodontitis and 9 about peri-implantitis. After title/abstract screening, 59 records were excluded because they were not pertinent, along with 1 book chapter and 2 abstracts only;therefore, 149 full-texts were assessed. According to the eligibility criteria for sources and study characteristics (Table 1), 1 commentary, 24 reviews, 19 animal and 18 in vivo/preclinical studies, besides 5 articles written in Chinese and Japanese languages, were not included. In addition, based on the inclusion/exclusion criteria on Population, Intervention, Comparison and Outcome(s), (Table 1), 9 studies involving participants with endodontic lesions, 9 including subjects with comorbidities and 12 comprising patients undergone periodontal treatment were also excluded; moreover, 44 studies, including interventions with local statins and 1 with high-dose systemically delivered statins, were not considered. 

Finally, 7 studies were included in the present systematic review, all of them concerning periodontitis and none regarding peri-implantitis.

### 3.2. Study Characteristics 

Each of the 7 included studies comprised a minimum of 70 periodontal subjects, at least 30 years old, without comorbidities potentially affecting their periodontal/peri-implant status, not under drugs affecting lipid nor bone metabolism, antibiotics and corticosteroids and not undergone periodontal treatment within the last 3 months. 

Atorvastatin was systemically administered in four studies [25,37,38,39], as well as simvastatin [25,37,40,41], while another unspecified statin was administered in one study [25]. Duration and dosing of statin intake werespecified in four studies [37,38,39,41], while only statin dosing was reported in three studies [38,39,41], and data on both duration and dosing of statin intakewere not described in the other three studies [25,40,42]. 

Statin intake was compared with no statin use in all the studies. Moreover, in four studies, the intervention was also compared with non-pharmacologic therapy, such as diet, combined or not with exercise, in hyperlipidemic subjects [38,40,41,42]. Statins were not compared with placebo in any of the selected studies. 

Clinical periodontal parameters around natural teeth were reported in all selected studies. Plaque Index (PI), or a similar parameter recording the presence of plaque [25], was evaluated in all the selected studies. Clinical Attachment Level (CAL) [38,39,40,41,42] and Periodontal Probing Depth (PPD) [25,38,40,41,42] were both measured in five studies, Gingival Index (GI) and Bleeding on Probing (BoP) were assessed in five[37,38,39,40] and three[25,41,42] studies, respectively, while the Community Periodontal Index was computed in one study [37]. Only two studies evaluated other periodontal parameters, such as gingival crevicular inflammatory mediators, specifically Interleukin (IL-)1B [39,42], IL-10 [42]and Myeloperoxidase (MPO) [42], and no studies considered either radiographic outcomes (bone loss), tooth loss due to periodontitis, nor the number of residual teeth. None of the included studies assessed periodontal parameters around implants.

Of the seven clinical studies included in the present systematic review, three were case–control and four were cross-sectional studies; no longitudinal nor randomized clinical trials were found to be compliant with the eligibility criteria. A complete description of the selected studies, regarding the source, participants, intervention, comparison andoutcomes and consideration(s) and conclusion(s) focusing on the PICO question, is reported in Table 2.

### 3.3. Risk of Bias within Studies 

Since the seven studies included in the present systematic review were all non-randomized studies, only the ROBINS-1 tool was currently employed, and the risk of bias within studies has been reported in Table 3.

**Results of individual studies.** 

Results concerning all outcomes considered in the current systematic review have been synthesized in a discursive way in Table 4.

## 4. Discussion

Discordant results were found in the literature on the putative positive effect exerted by systemically administered statins on clinical periodontal parameters around natural teeth and on gingival crevicular inflammatory mediators; no data, instead, were retrieved concerning alveolar bone loss nor clinical and radiographic periodontal parameters around dental implants.

The hypothesized bi-directional relationship between periodontitis and peri-implantitis, on the one side, and hyperlipidemia, on the other side, may rely on the shared etiologic factors, comprising genetic susceptibility, smoking, stress, altered immune and inflammatory response, and on the common pathogenic pathways [1]. Specifically, periodontal pathogens may gain access to the systemic circulation, colonize atheromatous plaques [43], and, along with their toxins and the proinflammatory cytokines from inflamed periodontal tissues, determine the so-called “systemic inflammation”, which is considered to be the key associating link between periodontitis and several systemic inflammatory and neoplastic diseases [1,8,10,11,12,13,14,44,45]. Consequently, the causal treatment of such disorders may positively affect both periodontal conditions around teeth and implants, as well as the lipidemic status [17,19]. In particular, systemic statins may be variously beneficial for periodontitis and peri-implantitis due to their main and pleiotropic effects. Indeed, combined with the hypolipidemic effects, statins have also shown anti-inflammatory and antioxidant activities, on the one side, and stimulating properties affecting endothelial function, angiogenesis and bone formation on the other side [46]. Noteworthy, in periodontal subjects, statins have been found able to reduce osteoclasts activity and bone resorption [39], to increase Interleukin- (IL-)10 in gingival crevicular fluid while decreasing IL-1B [42], together with IL-6 and IL-8 [31] and to inhibit the release of matrix metalloproteinase- (MMP-)1, MMP-2, MMP-3 and MMP-9 [25,47], finally lowering periodontal tissues inflammation and destruction.

The present systematic review, unlike the previous ones including studies on locally administered statins and/or on systemic statins as adjuncts to periodontal treatment [20,28,31], aimed to primarily evaluate the putative positive effect on untreated periodontitis and peri-implantitis of systemic statin therapy (≥1 month) alone. For such reasons, and due to the fact that periodontitis is linked to aging [48], studies involving participants younger than 30 years of age were excluded from the current analysis; similarly, studies including periodontally healthy subjects, and those reporting periodontal treatment, within the last 3 months or as an adjunct to statin administration, were not considered. In addition, studies comprising smokers and periodontal subjects with comorbidities were also excluded to eliminate the potential confounding due to the so-called periodontitis grade modifiers [49], specifically smoke and diabetes, as well as disorders and medications affecting lipid/bone metabolism and periodontal disease [50,51]. 

Periodontal parameters around natural teeth and dental implants were compared between statin users vs. non-statin users; no placebo use was reported in comparison with statin use in any of the included studies. Included studies described primarily simvastatin and secondarily atorvastatin intake, in compliance with the findings by Gu et al. showing that simvastatin represents 42% and atorvastatin 20% of all cholesterol-lowering medications, respectively [22]. 

Clinical periodontal parameters around natural teeth were recorded in all selected studies, although periodontal charting was variously performed, complicating results comparison. In addition, periodontal parameters were incompletely reported in many studies, with a lack of data describing and comparing periodontal status before vs. during/after statin use and in statins vs. non-statin users. Similarly, heterogeneous periodontal case definitions were applied in the included studies, different from the one introduced by the 2017 classification of periodontal and peri-implant diseases and conditions [7], and, consequently, staging and grading of periodontitis were not performed. In particular, the Community Periodontal Index, employedby Kadhim et al. [37] and by Fernández et al. [40], is considered to be able to detect the prevalence but not the severity of periodontal disease [40]. However, lower CAL values were reported in statin vs. non-statin users by Sayar et al. [41], who attributed such results to the anti-inflammatory effect exerted by statins, and by Cicek Ari et al., [42], although not reaching the statistical significance; conversely, opposite results were found by Poston et al. [52]. PPD values were improved by systemically delivered statins, as reported by Lindy et al. [24], Cicek et al. [42], Sayar et al. [41] and even by Poston et al. [50] but only in diabetic subjects, and by Fentoglu et al. [53] but only after more than 3 months of continuous statin intake; in addition, Lindy et al. [24] showed that also PIBI, combining the data on PPD, was 40% smaller in statin users when compared to non users. Many Authors [41,42,47,53]) detected a significant reduction in BOP values, likely linked to the anti-inflammatory effect exerted by statins, although Fentoglu et al. [53] described higher BOP values in the statin users than in non-users. Contrasting results were also reported for GI and PI values, resulted significantly lower in statin users in theSangwan et al. study [38], but not in the Cicek one [42]; Saxlin described a negative association between statinand periodontal statusamong subjects withplaque, although opposite results were found among subjects with no plaque [25]. 

Gingival crevicular IL-1 wassignificantly lower in statin compared to non-statin users [42,50], similarly to MPO [42] and IL-6 [51] levels, opposite to IL-10 ones [42]. Tooth loss may benefit from systemically delivered statins, which may exert a protective effect in periodontal subjects [19,25]. Radiographic periodontal parameters around natural teeth were not recorded nor reported in any of the included studies, even if bone loss may be positively affected by statin use, which has been hypothesized to reduce the risk of fracture and increase bone density [40]. 

Moreover, no study evaluating statins’ putative effect on peri-implant tissues was found, highlighting the need for a more comprehensive evaluation of the potential beneficial effect of systemic statins. Indeed, although peri-implantitis mainly shares ethio-pathogenic pathways with periodontitis [54], a certain association between peri-implant disease and hyperlipidemia has not been established, probably due to the paucity of studies investigating such a potential relationship [54].

In conclusion, discordant results were found in periodontal parameters, and the current lack of such data related to peri-implant tissues and to alveolar bone loss highlights the need for further studies on the topic, potentially paving the way for a more comprehensive approach to periodontitis and peri-implantitis management. Indeed, the validation of the beneficial effect provided by systemically delivered statins on periodontal and peri-implant tissues may direct recall scheduling, predict response to therapy and, therefore, guide treatment strategies of periodontal and peri-implant treatments in statin users.

## Figures and Tables

**Table 1:** Study eligibility: inclusion and non-inclusion criteria related to study sources, characteristics, population, intervention, comparison and outcome(s).

Study Eligibility	Inclusion Criteria	Non-Inclusion Criteria
Sources		
Databases	Electronic	Manual
Language	English language	Non-English language
Publication status	Published/in press/ahead of print	Submitted
Publication date	No restrictions	No restrictions
Text availability	Full-text access	Abstract-only
**Study Characteristics**		
Type	Clinical	In vitro
		Preclinical in vivo
Design	Randomized Clinical Trials (RCT)	Case reports
	Prospective	Case series
	Retrospective	Narrative reviews
	Case-control	Conference papers
	Cross-sectional	Oral communications
		Books and chapters
Sample size	≥70	<70
**Population**	Periodontal subjects	Periodontally healthy subjects
Age	≥30 years old	<30 years old
Gender	No restrictions	No restrictions
Characteristics	Smoker/no smokers	Pregnancy; lactation
Comorbidities	Hyperlipidemia	Systemic disease affecting lipid/bone metabolism or periodontal disease
	Cardiovascular disease	Medication-related osteonecrosis of the jaws
		Neoplastic disease
		Non-surgical/surgical periodontal treatment (within ≥3 months)
		Corticosteroids, antibiotics (within 3 months)
		Drugs affecting bone metabolism Radiations (head and neck)
Treatments	None	
**Intervention**	Statin use	No statin use
Type	Systemically delivered statins (any)	Locally delivered statins (any)
Duration	≥1 months	<1 months
Dosing	Low dose	High dose
**Comparison**	No statin use	Combined therapies
	Other types of statin	
	Placebo use	
**Outcome(s)**	Periodontal status	Endodontic–periodontal lesions
		Endodontic lesions
Clinical periodontal parameters	Clinical Attachment Level (CAL)	
	Periodontal Probing Depth (PPD)	
	Bleeding on Probing (BoP)	
	Gingival Index (GI)	
	Plaque Index (PI)	
	Tooth loss	
	Number of residual teeth	
Radiographic periodontal parameters	Bone loss	
Other periodontal parameters	Gingival crevicular (GC) Inflammatory mediators	

**Table 2:** Data extracted and collected from the studies included in the present systematic review. General information: first author, year and journal of publication; reference number; fundings. Characteristics of the study: design; participants (sample size, age and gender, smoking habit, comorbidities, treatments and procedures); intervention (statin type, dosing, duration and indication); comparison (no statine use, placebo); outcomes (clinical and radiographic periodontal parameters and Gingival crevicular Inflammatory mediators, assessment timepoints); consideration(s) and conclusion(s). Abbreviations: hyperlipidemics, “HL”; normolipidemics, “NL”; statin users, “S”; non-statin users, “NS”; diet, “D”; exercise, “E”; periodontally healthy, “Ph”; with gingivitis, “G”; with periodontitis, “P”; gingival crevicular, “GC”; relative risk, “RR”; *p*-value, “*p*”. Only statistically significant data concerning the outcomes and focusing on the PICO question have been reported.

Included Studies	Methods	Periodontal Parameters Evaluated	Outcome(s)	Conclusions and Considerations
**Author** **Year** **Journal** **Study design** **Reference […]** **Fundings**	**Participants** Subjects (n.) Age (y.o.) Male/Female Smoking habit **Intervention** Statin type Dosing Duration **Comparison** No drug therapy (hyperlipidemics)/different statin (hyperlipidemics)/ No statin (normolipidemics) **Procedure(s)** Any	**Clinical** CAL PPD BoP Gingival Index (GI) Plaque Index (PI) Tooth loss Teeth n. **Radiographic** Bone loss **Gingival crevicular (GC)** Inflammatory mediators	**Statistically significant** **(*p* < 0.05)**	
**Cicek Ari** 2016 Inflam **Case–control study** **[42]** The study was financially supported by the Hacettepe University Scientific Research Projects Coordination Unit	**Participants** Subjects n. 127 Non-smokers Classified as: HL-S 20M/33F Sub-classified as: - “Ph” (n.14) Age (53.0 ± 13.08 y.o.) - G (n. 17) Age (55.47 ± 11.59 y.o.) - P” (n.22) Age (58.86 ± 6.93 y.o.) HL-D 8M/18F Sub-classified as: - Ph (n.14) Age (53.0 ± 13.08 y.o.) - G (n. 13) Age (46.08 ± 12.38 y.o.) - P (n.13) Age (53.69 ± 9.8 y.o.) NL-NS 17 M/31F Sub-classified as: - Ph (n.18) Age (27.7 ± 3.6 y.o.) - G (n. 15) Age (29.67 ± 11.5 y.o.) - P (n.15) Age (41.40 ± 8.8 y.o.) **Intervention** Statin type: n.a. Dosing: n.a. Duration: n.a. **Comparison** No drug therapy (diet)/no statin **Procedure(s)** - Periodontal exam - Gingival crevicular fluid (GCF) sampling and analysis for IL-1B, IL-10, MPO - Blood sampling and analysis of serum triglycerides, total cholesterol, LDL, HDL and fasting plasma glucose	**Clinical** CAL PPD PI GI BOP **Gingival crevicular** IL-1B IL-10 MPO	PPD (mm): HL-S-P (2.30 ± 0.85) < HL-D-P (2.76 ± 1.01) < NL-NS-P (3.03 ± 0.58) *p* = 0.012 GCF IL-1B (pg/mL): HL-S-P (72.43 ± 65.19) < NL-NS-P (90.41 ± 67.78) < HL-D-P (142.08 ± 79.82) *p* < 0.05	PPD values of hyperlipidemic statin users with periodontitis were significantly lower compared to normolipidemics with periodontitis IL-1B levels of hyperlipidemic statin users with periodontitis were significantly lower compared to hyperlipidemics on diet with periodontitis Statin use decreased the IL-1B and MPO levels and enhanced IL-10 in GCF **Statins may decrease periodontal inflammation and periodontitis progression**
**Fernandez** 2014 J Perio **Cross-sectional study** **[40]** The study was partially funded by Research GroupCTS-583 of the Andalusian Regional Government	**Participants** Subjects n. 73 Classified as: HL-S-Ph/G/P (n.29) Age (63.1 ± 8.9 y.o.) 18M/11F Smokers: 10.3% HL-D-Ph/G/P (n.28) Age (52.7 ± 9.0 y.o.) 10M/8F Smokers: 21.5% NL-NS-Ph/G/P (n.16) Age (46.3 ± 10.7 y.o.) 8M/8F Smokers: 37.5% **Intervention** Statin type: simvastatin Dosing: n.a. Duration: 3–132 months **Comparison** No drug therapy (diet)/no statin **Procedure(s)** - Periodontal exam - Blood sampling and analysis of serum triglycerides, total cholesterol, LDL, HDL and fasting plasma glucose, C-reactive protein, erythrocyte sedimentation rate and bone metabolismmarkers (osteoprotegerin, osteocalcin, procollagen type I N-terminal propeptide and C-terminal telopeptide of type I collagen)	**Clinical** CAL PPD PI GI	CAL (mm): HL-S-P (2.7 ± 1.2) < NL-NS-P (3.0 ± 1.6) *p* = 0.05	CAL values of hyperlipidemic statin users with periodontitis were significantly lower compared to normolipidemics with periodontitis **Periodontal status was similar betweenhyperlipidemic statin users with periodontitis and normolipidemics with periodontitis, but worse in hyperlipidemics on diet with periodontitis**
**Kadhim** 2019 Dental Hypothesis **Cross-sectional study** **[37]** No fundings	**Participants** Subjects n. 74 Age (40–69 y.o.) M/F: n.a. Smokers (40/74) Classified as: HL-S-P (n.40) HL-NS-P (n.34) NL-NS-Ph (n.30) **Intervention** Statin type: simvastatin (45.00%) /atorvastatin (55.00%) Dosing: n.a. Duration: > 6 months **Comparison** No statin **Procedure(s)** - Periodontal exam - Blood sampling and analysis of serum triglycerides, total cholesterol, LDL, HDL and of inflammatory biomarkers (C-reactive protein, Interleukin-6, Tumor Necrosis Factor- a, malondialdehyde) - Blood pressure measurement	Clinical Community Periodontal Index Teeth n. PI GI	Community Periodontal Index: HL-S-P < HL-NS-P *p* < 0.01 Teeth (n.): HL-NS-P + HL-S-P (15.73 ± 1.58) <NL-NS-Ph (22.28 ± 1.63) *p* = 0.001 PI: HL-S-P < HL-NS-P *p* < 0.01 GI: HL-S-P < HL-NS-P *p* < 0.01 Smokers: NL-NS-Ph (n.7) < HL-S-P + HL-NS-P (n.33) *p* = 0.04	Hyperlipidemic statin users and non-users with periodontitis had higher levels of smoking status and number of residual teeth compared with normolipidemic nonperiodontal subjects **Atorvastatin** **and** **simvastatin** **produced** **comparable effects in the reduction of PI, GI and Community** **Periodontal Index** **in hyperlipidemic statin users with periodontitis**
**Sangwan** 2013 Eur J Dent **Cross-sectional study** **[38]** No fundings	**Participants** Subjects n. 140 Non-smokers Classified as: HL-S-P (n.50) Age (45.62–9.90 y.o.) 29M/21F HL-NS/D/E-P (n.44) Age (41.34–10.02 y.o.) 25M/19F NL-NS-P (n.46) Age (42.54–9.91 y.o.) 22M/24F **Intervention** Statin type: atorvastatin Dosing: 20 mg/die Duration: ≥ 3 months **Comparison** No drug therapy (diet and exercise)/no statin **Procedure(s)** - Periodontal exam - Blood sampling and analysis of serum triglycerides, total cholesterol, LDL and HDL levels	**Clinical** CAL PPD Teeth n. GI PI	CAL (mm): NL-NS-P (3.64 ± 0.86) < HL-S-P + HL-NS-P (4.12 ± 1.26) *p* = 0.047 PPD (mm): NL-NS-P (2.78 ± 0.53) < HL-S-P + HL-NS-P (3.24 ± 0.89) *p* = 0.003 Teeth n.: NL-NS-P (26.76 ± 1.58) < HL-S-P + HL-NS-P (25.18 ± 2.99) *p* = 0.002 PPD (mm): HL-S-P (3.00 ± 0.81) < HL-NS/D/E-P (3.52 ± 0.90) *p* = 0.001 GI: HL-S-P (1.44 ± 0.30) < HL-NS/D/E-P (1.59 ± 0.33) *p* = 0.022	Hyperlipidemic statin users and non-users with periodontitis had higher CAL and PPD values and number of lost teeth compared with normolipidemic periodontal subjects Hyperlipidemic non-statin users with periodontitis had higher GI values compared with hyperlipidemic statin users with periodontitis and normolipidemic periodontal subjects **Statins (atorvastatin) may positively affect gingival inflammation**
**Sayar** 2016 Oral dis **Cross-sectional study** **[41]** No fundings	**Participants** Subjects n. 150 Non-smokers Classified as: HL-S-P (n.50) Age (47.10 ± 5.61 y.o.) 28M/22F HL-NS/D/E-P (n.50) Age (46.98 ± 5.79 y.o.) 21M/29F NL-NS-P (n.50) Age (45.42 ± 6.61 y.o.) 24M/26F **Intervention** Statin type: simvastatin Dosing: 40 mg/die Duration: ≥ 3 months **Comparison** No drug therapy (diet and exercise)/no statin **Procedure(s)** - Periodontal exam - Blood sampling and analysis of serum triglycerides, total cholesterol, LDL and HDL levels	**Clinical** CAL PPD BoP PI	CAL (mm). NL-NS-P (1.03 ± 0.35) < HL-S-P (1.83 ± 0.67) < HL-NS-P (2.00 ± 0.72) *p* = 0.0001 PPD (mm): NL-NS-P (2.24 ± 0.35)< HL-S-P (2.76 ± 0.39) < HL-NS-P (3.15 ± 0.46) *p* = 0.0001 PI: HL-S-P (2.32 ± 0.54) < HL-NS-P (2.67 ± 0.45) *p* = 0.005	Hyperlipidemic statin users and non-users with periodontitis had higher PD and CAL values compared with normolipidemic periodontal subjects Hyperlipidemic statin users with periodontitis had lower PI values compared with hyperlipidemic non-statin users with periodontitis and normolipidemic periodontal subjects **Statins (simvastatin) may exert an anti-inflammatory effect**
**Saxlin** 2009 J Clin Perio **Cross-sectional study** **[25]** The study was partly supported by the Finnish Dental Association and by the Finnish Dental Society Apollonia	**Participants** Subjects n. 2032 Age (40–69 y.o.) 43,4M/56,5F Non-smokers Classified as: HL-S-P (n.134) Age (58,4 y.o.) 53M/47F Sub-classified as: - Simvastatin users (n.58) - Atorvastatin users (n.38) - Other statin type users (n.38) NL-NS-P (n.1898) Age (52,1 y.o.) 43M/57F **Intervention** Statin type: simvastatin/atorvastatin/other statin Dosing: n.a. Duration: n.a. **Comparison** No statin/Simvastatin/Atorvastatin/Other statin **Procedure(s)** - Periodontal exam	**Clinical** PPD (N. teeth with deepened periodontal pockets ≥ 4 mm; N. teeth with deeper periodontal pockets ≥ 6 mm) BoP Presence of Plaque	N. teeth with deepened periodontal pockets ≥ 4 mm: HL-S-P with gingival bleeding (RR 0.9, 95% CI 0.7–1.1) > NL-NS-P N. teeth with deeper periodontal pockets ≥ 6 mm: HL-S-P with gingival bleeding (RR 0.6, 95% CI 0.3–1.1) > NL-NS-P	Beneficial effect of statins was seen in hyperlipidemic statin users with periodontitis with dental plaque or gingival bleeding. Among hyperlipidemic statin users with periodontitis but no dental plaque nor gingival bleeding; statins were associated with deepened periodontal pockets **Statins may have beneficial effects on the periodontium** **Statin effect may be dependent on the inflammatory conditions of the periodontium**
**Suresh** 2013 Indian J Pharmacol **Cross-sectional** **[39]** No fundings	**Participants** Subjects n. 30 Age (40–60 y.o.) 16M/14F Non-smokers Classified as: HL-S-P (n.15) HL-NS-P (n.15) **Intervention** Statin type: atorvastatin Dosing: 20 mg/die Duration: ≥ 6 months **Comparison** No statin **Procedure(s)** - Periodontal exam - Gingival crevicular Fluid (GCF)—Sampling and analysis for IL-1B	**Clinical** CAL PI GI **Gingival crevicular** IL-1B	GCF IL-1B (pg/mL): HL-S-P (180.73 ± 32.15) < HL-NS-P(308.20 ± 27.73) *p <* 0.001 CAL, PI and GI: HL-S-P = HL-NS-P (selection criteria) Specifically, CAL (mm): HL-S-P (4.1) = HL-NS-P (3.9); PI: HL-S-P (2.6 ± 0.51) = HL-NS-P (2.4 ± 0.51) GI:HL-S-P (2.55 ± 0.35) = HL-NS-P (2.65)	Hyperlipidemic statin users with periodontitis had lower levels of GCF IL-1Bcompared to hyperlipidemic statin users with periodontitis and similar CAL, PI and GI values **Atorvastatin may exert an anti-inflammatory effect on chronic periodontitis**

**Table 3:** Risk of bias of non-randomized clinical trials, assessed through the ROBINS-I tool [36], is designated as “Low”, “High” and “Unclear”.

Study	Confounding	Selection of Participants	Classification of Interventions	Deviations from Intended Interventions	Measurement of Outcomes	Missing Data	Selection of the Reported Result
Cicek Ari, 2016	Unclear	Low	Low	Low	High	Low	Low
Fernandez, 2014	Unclear	Low	Low	Low	High	Unclear	Unclear
Kadhim, 2019	Unclear	Low	Low	Low	High	Unclear	Unclear
Sangwan, 2013	Unclear	Low	Low	Low	High	Low	Low
Sayar, 2016	Unclear	Low	Low	Low	High	Low	Low
Saxlin, 2009	Unclear	Low	Low	Low	High	Low	Low
Suresh, 2013	Unclear	Low	Low	Low	High	Unclear	Unclear

**Table 4:** Synthesis of the outcomes evaluated.

Periodontal Parameter(s)	Author, Year	Study Design	Main Result(s)
CAL	Cicek Ari, 2016	Case–control	CAL values were lower in statin users but did not reach statistically significant difference between statin and non-statin users
	Fernandez, 2014	Cross-sectional study	CAL values were lower in statin users compared to non-statin users
	Sangwan, 2013	Cross-sectional study	CAL values were similar between statin and no statin users
	Sayar, 2016	Cross-sectional study	CAL values were higher in non-statin compared to statin users
	Suresh, 2013	Cross-sectional study	CAL values were similar in statin and non-statin users
PPD	Cicek Ari, 2016	Case/control	PPD values were lower in statin users compared to normolipidemic non-statin users
	Sangwan, 2013	Cross-sectional study	PPD values were lower in statin compared to hyperlipidemic non-statin users
	Sayar, 2016	Cross-sectional study	PPD values were lower in statin compared to non-statin users
BoP	Cicek Ari, 2016	Case/control	BoP values were lower in statin compared to non-statin users
	Saxlin, 2009	Cross-sectional study	BoP values were higher in statin compared to non-statin users
	Sayar, 2016	Cross-sectional study	BoP values were similar in statin compared to non-statin users
GI	Cicek Ari, 2016	Case–control	Statins use did not improve GI
	Kadhim, 2019	Cross-sectional study	GI values were lower in statin compared to non-statin users
	Suresh, 2013	Cross-sectional study	CAL values were similar in statin and non-statin users
PI	Kadhim, 2019	Cross-sectional study	PI values were lower in statin compared to non-statin users
	Sangwan, 2013	Cross-sectional study	PI values were similar in statin and non-statin users
	Sayar, 2016	Cross-sectional study	PI values were lower in statin compared to non-statin users
	Suresh, 2013	Cross-sectional study	PI values were similar in statin and non-statin users
Tooth loss	Saxlin, 2009	Cross-sectional study	Statin use was associated with decreased tooth loss among subjects with chronic periodontitis
Residual teeth	Sangwan, 2013	Cross-sectional study	The number of residual teeth was negatively associated with TGs
IL-1B (crevicular)	Cicek Ari, 2016	Case–control	GCF IL-1b levels were lower in statin compared to non-statin users
	Suresh, 2013	Cross-sectional study	GCF IL-1B levels were lower in statin compared to non-statin users
IL-10 (crevicular)	Cicek Ari, 2016	Case–control	GCF IL-10 levels were higher in statin compared to non-statin users
MPO (crevicular)	Cicek Ari, 2016	Case–control	GCF MPO were lower in statin compared to non-statin users

## Data Availability

Medline (PubMed) and Scopus databases.
